# Ghrelin and Its Receptors in Gilthead Sea Bream: Nutritional Regulation

**DOI:** 10.3389/fendo.2018.00399

**Published:** 2018-07-30

**Authors:** Miquel Perelló-Amorós, Emilio J. Vélez, Jaume Vela-Albesa, Albert Sánchez-Moya, Natàlia Riera-Heredia, Ida Hedén, Jaume Fernández-Borràs, Josefina Blasco, Josep A. Calduch-Giner, Isabel Navarro, Encarnación Capilla, Elisabeth Jönsson, Jaume Pérez-Sánchez, Joaquim Gutiérrez

**Affiliations:** ^1^Department of Cell Biology, Physiology and Immunology, Faculty of Biology, University of Barcelona, Barcelona, Spain; ^2^Fish Endocrinology Laboratory, Department of Biological and Environmental Sciences, University of Gothenburg, Gothenburg, Sweden; ^3^Nutrition and Fish Growth Endocrinology, Institute of Aquaculture Torre de la Sal (CSIC), Castellón, Spain

**Keywords:** ghrelin, GHSR1a, growth hormone, IGF-1, fasting and refeeding

## Abstract

Ghrelin is involved in the regulation of growth in vertebrates through controlling different functions, such as feed intake, metabolism, intestinal activity or growth hormone (Gh) secretion. The aim of this work was to identify the sequences of *preproghrelin* and Ghrelin receptors (*ghsrs*), and to study their responses to different nutritional conditions in gilthead sea bream (*Sparus aurata*) juveniles. The structure and phylogeny of *S. aurata preproghrelin* was analyzed, and a tissue screening was performed. The effects of 21 days of fasting and 2, 5, 24 h, and 7 days of refeeding on plasma levels of Ghrelin, Gh and Igf-1, and the gene expression of *preproghrelin, ghsrs* and members of the Gh/Igf-1 system were determined in key tissues. *preproghrelin* and the receptors are well conserved, being expressed mainly in stomach, and in the pituitary and brain, respectively. Twenty-one days of fasting resulted in a decrease in growth while Ghrelin plasma levels were elevated to decrease at 5 h post-prandial when pituitary *ghsrs* expression was minimum. Gh in plasma increased during fasting and slowly felt upon refeeding, while plasma Igf-1 showed an inverse profile. Pituitary *gh* expression augmented during fasting reaching maximum levels at 1 day post-feeding while liver *igf-1* expression and that of its splice variants decreased to lowest levels. Liver Gh receptors expression was down-regulated during fasting and recovered after refeeding. This study demonstrates the important role of Ghrelin during fasting, its acute down-regulation in the post-prandial stage and its interaction with pituitary Ghsrs and Gh/Igf-1 axis.

## Introduction

Ghrelin is a peptide hormone secreted mainly by the stomach in vertebrates, but also detected in many other tissues (e.g., intestine, heart, pancreas, and especially pituitary and brain). Ghrelin is synthesized as Preproghrelin, and the mature peptide varies between 12 and 28 amino acids, depending on species and form of Ghrelin, but it shows high sequence homology across vertebrates, including fish (1). Since its discovery, Ghrelin has been involved in many physiological processes like the regulation of feed intake, adiposity, growth, energy and glucose metabolism, intestinal motility and digestive enzymes activity, among others (2). The first characterization of Ghrelin in a fish species, the goldfish (*Carassius auratus*), was done by Unniappan et al. (3). Later, Kaiya et al. (4) reviewed its function in non-mammalian vertebrates and recently, different publications have investigated its role in other fish species (5–12), but very little is known about this hormone in gilthead sea bream (*Sparus aurata*) (13).

Ghrelin functions through binding to its receptors, which are also known as the growth hormone secretagogue receptors (Ghsrs). The Ghsrs are a family of transmembrane G-protein coupled receptors, and the Ghsr1a isoform, discovered a few years before Ghrelin (14), is known as the active form. An alternative splice variant named Ghsr1b, was also described by the same authors, but its structure lacks two transmembrane domains leading to the impossibility of this isoform to initiate intracellular signaling. Since the discovery of these two receptors, the Ghsrs family has been widely studied and other numerous isoforms (splice variants and paralogues) have been described in vertebrates (15, 16).

*ghsrs* mRNA is found in many tissues, including brain, stomach, intestine, and especially pituitary gland. The high expression levels detected in the pituitary in vertebrates confirms the role of Ghsrs in the regulation of growth hormone (Gh) production (17). Gh is one of the key elements of the Gh/insulin-like growth factor-1 (Igf-1) axis, which is the main regulator of growth in vertebrates. Depending on factors such as nutritional state, Gh can directly stimulate anabolic or catabolic processes by binding to the Gh receptors (Ghrs). Moreover, systemic Gh mainly acts in the liver, where it stimulates the production of Igf-1. This growth factor in turn acts in many peripheral tissues stimulating growth-related processes (18). Thus, most of the physiological peripheral roles of Ghrelin appear to be mediated indirectly by the modulation of Gh release (19). In addition, Ghrelin has been described to have an important role in the hypothalamus in mammals, where it acts on different *ghsrs*-expressing cell populations, leading to enhanced expression and release of orexigenic neuropeptides like neuropeptide Y and Agouti-related peptide, hence stimulating appetite in most vertebrates, including diverse fish species (20). Moreover, it has been recently reported, at least in mammals, that Ghrelin acts over the hypothalamic Gh releasing hormone neurons (21). Although there is a controversy on how the different forms of Ghrelin (acylated and unacylated) cross the blood brain barrier to exert this role (22), adding another complex level of regulation.

Fish are capable to resist long fasting periods (23) and the Gh/Igf-1 system, displays interesting changes to adjust metabolism and growth to nutrient supply. Ghrelin in its double role as a hunger hormone and Gh secretion regulator should play an important role in fasting and refeeding physiology, although these aspects are poorly known in fish, especially in gilthead sea bream (13, 24).

The objective of the present work was to identify and characterize Ghrelin and its receptors by analyzing sequences, phylogeny and gene expression through a tissue screening, and to study their responsiveness upon fasting and refeeding in relation with the Gh/Igf-1 axis in gilthead sea bream juveniles.

## Materials and methods

### Fish maintenance and distribution

Gilthead sea bream juveniles (initial body weight 50 ± 3 g; length 15.3 ± 0.68 cm) were obtained from a commercial hatchery (Piscimar, Borriana, Spain) and reared in the facilities of the Faculty of Biology. Forty-two fish were randomly distributed in six 200 L seawater tanks (7 fish/tank) and some extra fish for tissue screening were kept in another 200 L tank. Fish were kept in a seawater recirculation system at a constant temperature of 23 ± 1°C and at 12 h light/12 h dark photoperiod through the whole experiment. During the acclimation period (2 weeks), fish were fed to apparent satiety twice a day with a commercial diet (Optibream, Skretting, Burgos, Spain). This study was carried out in accordance with the recommendations of the EU, Spanish and Catalan Government-established norms and procedures. The protocol was approved by the Ethics and Animal Care Committee of the University of Barcelona (permit numbers CEEA 110/17 and DAAM 9488).

### Experimental design

After acclimation, a period of 21 days of fasting and 7 days of refeeding was designed, according to previous experience (25). During the refeeding period, fish were fed once a day to apparent satiety. Samplings were made at the beginning and end of the fasting period (−21 and 0 days, respectively), and at 2, 5, 24 h and 7 days upon refeeding. The −21 days, 24 h and 7 days samplings were made just before feeding, representing one day fasting. The day 0 sampling was done at the same time of the day, and fish were fed right after to start the refeeding. In each sampling, 6 fish were first anesthetized with MS-222 (0.08 g/L), and once blood was extracted, were sacrificed by an overdose of MS-222 (>0.1 g/L). Then, brain, pituitary, liver and stomach were dissected and stored in liquid nitrogen. Before sacrifice, body weight, body length (standard), and liver and viscera weight were measured to calculate distinct biometric indexes: condition factor (CF), hepatosomatic index (HSI), and viscerosomatic index (VSI).

Additionally, 3 fish were sacrificed and sampled for 17 distinct tissues and/or organs. RNA was obtained from tissue samples (30–100 mg) or from the whole pituitary gland and brain with 1 mL of TRI Reagent Solution (Applied Biosystems, Alcobendas, Spain) and reverse transcribed following the procedures previously described (26). Briefly, 1 μg of RNA was treated with DNase I (Life Technologies, Alcobendas, Spain) following the manufacturer's instructions to remove genomic DNA. After DNase treatment, retrotranscription was performed using the Transcriptor First Strand cDNA Synthesis Kit (Roche, Sant Cugat del Vallès, Spain) for 10 min at 25°C, 60 min at 50°C and 5 min at 85°C. Samples were immediately stored at −20°C for further analysis.

### *Preproghrelin* and *ghsrs* characterization

Primers for the amplification of the complete codifying sequences of *preproghrelin, ghsr1a* and *ghsr1b* were designed using Primer3Plus software (27) with the nucleotide sequences obtained from the Nutrigroup-IATS nucleotide database of gilthead sea bream at http://www.nutrigroup-iats.org/seabreamdb (28, 29)]. The three sequences are deposited in GenBank (NCBI) under accession numbers: MG570187 for *preproghrelin*; MG570188 for *ghsr1a*, and MG570189 for *ghsr1b*. Sequences specificity was confirmed by PCR amplification of transcribed RNA samples from the tissue screening that were run on an agarose gel for size verification.

A multiple Preproghrelin sequence alignment was performed using the default settings of the MAFFT tool online (server) version (http://mafft.cbrc.jp/alignment/server/). The phylogeny was inferred using the JTT + G + I model substitution method and an unrooted tree was constructed using the MEGA X software with a bootstrapping value of 1,000. Previously, using the same software, a test was performed to determine which substitution model was the best for our data (data not shown). Unequivocal identity of *ghsr1a* and *ghsr1b* was verified by Blast and BlastX searches, as well as by transmembrane domain analysis by means of TMHMM transmembrane helixes prediction program (http://www.cbs.dtu.dk/services/TMHMM-2.0).

### Ghrelin, Gh and Igf-1 plasma levels

Plasma levels of acylated Ghrelin were measured using the Ghrelin N- radioimmunoassay (RIA) protocol originally described by Hosoda et al. (30) and modified by Jönsson et al. (7) with the exception that plasma was not extracted, just quickly centrifuged (1,000 rpm, 1 min) before pipetting to the RIA tubes, and iodinated human Ghrelin (NEX388010UC, PerkinElmer, USA) was applied as tracer. Anti-rat Ghrelin [1-11] antisera, which specifically recognizes the conserved n-octanoylated Ser3 epitope on Ghrelin, was used at a final dilution of 1:500000 (gift from Dr. Hiroshi Hosoda, Japan). Standard was made using synthetic rainbow trout acylated Ghrelin (Peptide institute, Japan).

All samples were assayed in duplicate and included in one assay. The Ghrelin RIA was validated for gilthead sea bream, and the slopes of the standard curve and of a serial dilution of plasma samples were parallel (Supplementary Figure 1). Plasma levels of Gh and Igf-1 were measured by corresponding RIAs, as previously described (31, 32).

### Gene expression

The mRNA transcript levels were examined by quantitative real-time PCR (qPCR) according to the requirements of *MIQUE guidelines* (33) in a CFX384™ Real-Time System (Bio-Rad, El Prat de Llobregat, Spain). All reactions were performed in the conditions previously described (26). The primers used are listed in Table 1. To amplify the two *ghsrs* the forward primer was designed in a common region, and the reverse primer for *ghsr1a* in a region overlapping exon 1 and 2, and for *ghsrb1* in a region including the differential nucleotides at the end of translation and the 3′-UTR. In addition, elongation factor 1 alpha (*ef1a*), ribosomal protein S18 (*rps18*) and *b-actin* (only in brain) were analyzed and served as reference genes in order to calculate the relative expression of the target genes (34). Both, reference genes stability and relative expression calculation were determined with the Bio-Rad CFX Manager Software (v2.1).

**Table 1:** Sequences, melting temperatures (Tm) and GenBank accession numbers of the primers used for qPCR.

**Primer list (*Sparus aurata*)**			
**Gene**	**Sequence (5′-3′)**	**Tm (°C)**	**Accession Number**
*ef1a*	**F:** CTTCAACGCTCAGGTCATCAT	60	AF184170
	**R:** GCACAGCGAAACGACCAAGGGGA		
*rps18*	**F:** GGGTGTTGGCAGACGTTAC	60	AM490061.1
	**R:** CTTCTGCCTGTTGAGGAACCA		
*b-actin*	**F:** TCCTGCGGAATCCATGAGA	60	X89920
	**R:** GACGTCGCACTTCATGATGCT		
*preproghrelin*	**F:** CCCGTCACAAAAACCTCAGAAC	60	MG570187
	**R:** TTCAAAGGGGGCGCTTATTG		
*ghsr1a*	**F:** GTCGGCGGCTGTGGCAAAGA **R:** GGCCAACACCACCACCACCAAC	60	MG570188
*ghsr1b*	**F:** CGCACACGCATAACTTTGTC	60	MG570189
	**R:** GAGGAGGATGAGCAGGTGAA		
*gh*	**F:** GCCCCATCGACAAGCACG	60	FJ827496
	**R:** GAGTCTACATTTTGCCACCGTCAG		
*ghr-1*	**F:** ACCTGTCAGCCACCACATGA	60	AF438176
	**R:** TCGTGCAGATCTGGGTCGTA		
*ghr-2*	**F:** GAGTGAACCCGGCCTGACAG	60	AY573601
	**R:** GCGGTGGTATCTGATTCATGGT		
*igf-1a*	**F:** AGGACAGCACAGCAGCCAGACAAGAC	60	AY996779
	**R:** TTCGGACCATTGTTAGCCTCCTCTCTG		
*igf-1ab*	**F:** AGTCATTCATCCTTCAAGGAAGTGCATCC	60	EF688015
	**R:** TTCGGACCATTGTTAGCCTCCTCTCTG		
*igf-1abc*	**F:** ACAGAATGTAGGGACGGAGCGAATGGAC	60	EF688016
	**R:** TTCGGACCATTGTTAGCCTCCTCTCTG		
*igfbp1a*	**F:** AGTGCGAGTCCTCTCTGGAT	60	KM522771
	**R:** TCTCTTTAAGGGCACTCGGC		
*igfbp2a*	**F:** CGGGCTGCTGCTGACATACG	60	AF377998
	**R:** GTCCCGTCGCACCTCATTTG		
*igfbp4*	**F:** TCCACAAACCAGAGAAGCAA	60	F5T95CD
	**R:** GGGTATGGGGATTGTGAAGA		02JMZ9K
*igfbp5b*	**F:** TTTCTCTCTCGGTGTGC	60	AM963285
	**R:** TCAAGTATCGGCTCCAG		
*igf-rb*	**F:** GCTAATGCGAATGTGTTGG	55	KT156847
	**R:** CGTCCTTTATGCTGCTGATG		

### Statistical analyses

Data was analyzed using IBM SPSS Statistics 22 and are showed as mean ± standard error of the mean (SEM). Normality and homogeneity of variances were tested with Shapiro-Wilk Test and Levene's, respectively. When data did not follow a normal distribution or did not have homoscedasticity, it was converted by logarithm transformation. Differences among groups were tested by one-way analysis of variance (ANOVA) followed by Tukey HSD or LSD, as *post-hoc* tests. In case of no homoscedasticity, the non-parametric Kruskal-Wallis test was used with the Dunnett's T3 as *post-hoc*. The confidence interval for all analyses was set at 5%.

## Results

### Preproghrelin and ghsrs characterization

**Figure 1:**
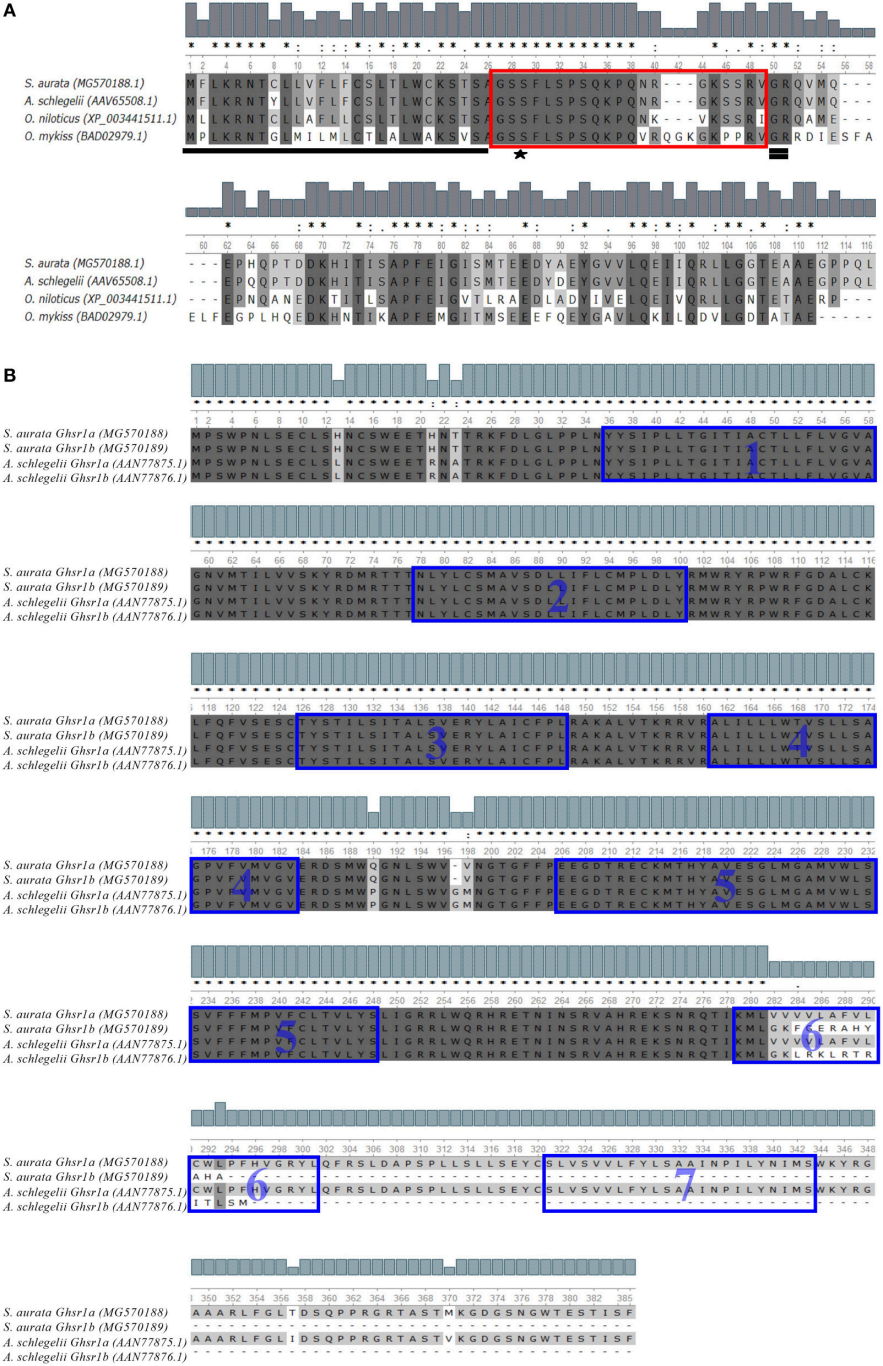
**(A)** Multiple alignment of the Preproghrelin amino acid sequences corresponding to members of sparidae perciformes (*S. aurata* and *A. schlegelii*), cichlidae perciforme (*O. niloticus*) and salmoniformes (*O. mykiss*). From N- to C-terminal, the signal peptide (underlined), the mature Ghrelin (red boxed) and C-terminal Proghrelin peptide (rest of the sequence) are highlighted. Moreover, the acylation target Ser3 residue (starred) and the GlyArg amidation and cleavage signal (double underlined) are identified and conserved. **(B)** Amino acid alignment of the translated sequences of *S. aurata* Ghsr1a and Ghsr1b with their respective orthologs of the sparidae perciforme (*A. schlegelii*). Predicted transmembrane domains are blue boxed. Percentage of identity is indicated in grey scale. “^*^” indicates positions which have a single, fully conserved residue; “:” indicates conservation between groups of strongly similar properties - scoring > 0.5 in the Gonnet PAM 250 matrix and “.” indicates conservation between groups of weakly similar properties - scoring ≤ 0.5 in the Gonnet PAM 250 matrix.

Translation of *preproghrelin* nucleotide sequence (907 nucleotide in length) resulted in a 107 amino acid sequence that presented 97% identity with that of another sparid, the blackhead sea bream (*Acanthopagrus schlegelii*), as the most significant result in a BlastX search. The predicted sequence of gilthead sea bream Preproghrelin contained the conserved N-terminal signal peptide (26 amino acids), that yields Proghrelin after cleavage. In the Proghrelin region, the sequence contained the characteristic Ser3 residue, which is the octanonylation target, as well as the GlyArg amidation and cleavage site to obtain the N-terminal mature Ghrelin (20 amino acids) and the C-terminal Proghrelin peptide (Figure 1A).

The nucleotide sequences of *ghsr1a* and *ghsr1b* (1708 and 1793 nucleotide in length, respectively) encoded for 384 (Ghsr1a) and 292 (Ghsr1b) amino acids sequences that shared a 98% of identity with their respective orthologs in the blackhead sea bream (35). In the same way, the TMHMM transmembrane helixes program predicted the presence of the characteristic seven transmembrane domains in Ghsr1a, whereas Ghsr1b did not retain the last two due to alternative gene splicing (Figure 1B).

**Figure 2:**
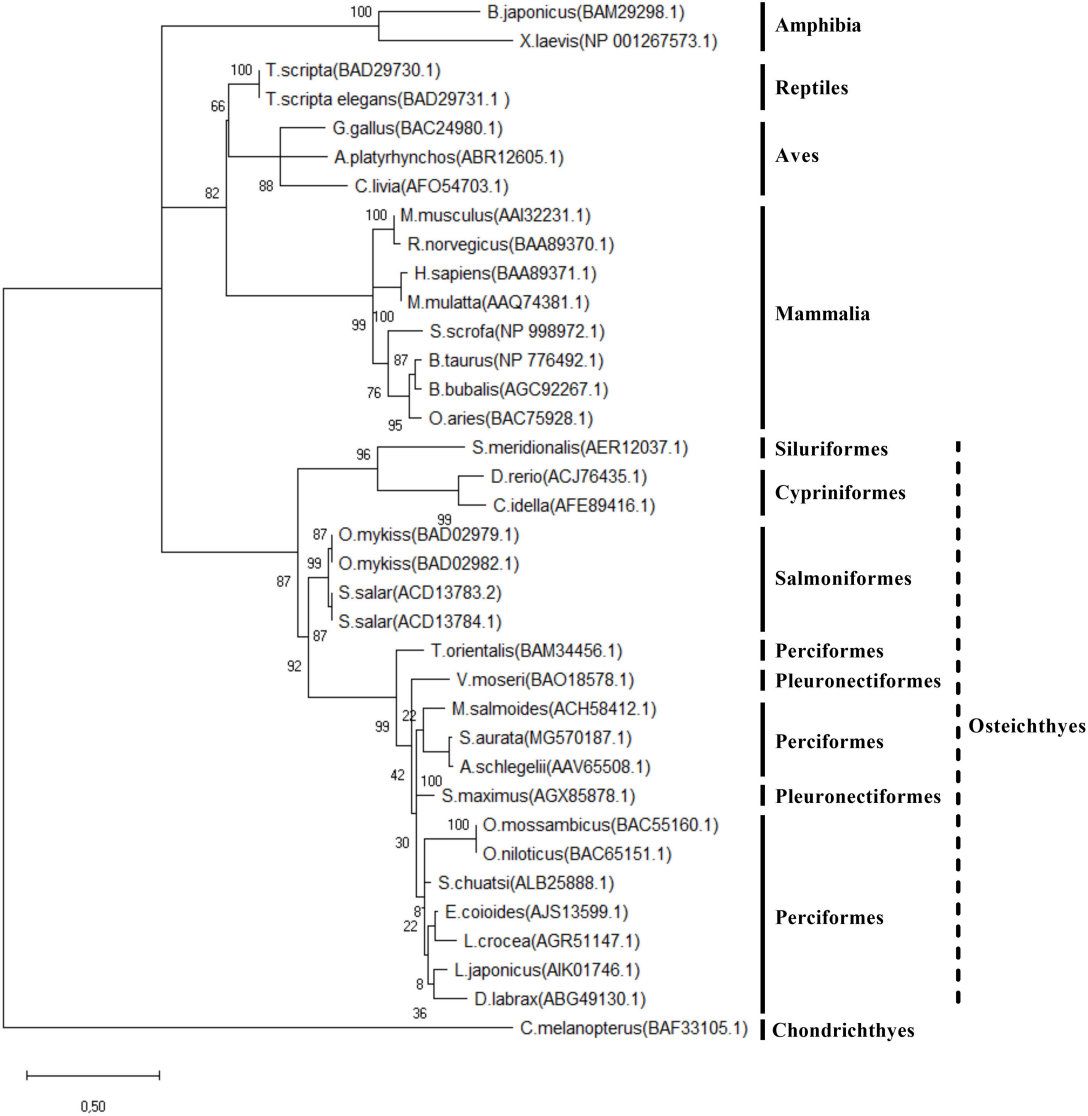
Phylogenetic analysis (unrooted tree) of Preproghrelin among different vertebrates. Multiple alignment was performed using the default settings of the MAFFT tool online (server) version (http://mafft.cbrc.jp/alignment/server/) and a phylogenetic tree by Maximum Likelihood phylogeny was built with the MEGA X tool using the JTT + G + I substitution model. *B. japonicus* (*Bufo japonicus*)*, X. laevis* (*Xenopus laevis*)*, G. Gallus* (*Gallus gallus*), *A. platyrhynchos* (*Anas platyrhynchos*), *C. livia* (*Columba livia*) *T. scripta* (*Trachemys scripta*), *M. musculus* (*Mus musculus*), *R. norvegicus* (*Rattus norvegicus*), *H. sapiens* (*Homo sapiens*), *M. mulatta* (*Macaca mulatta*), *S. scrofa* (*Sus scrofa*), *O. aries* (*Ovis aries*), *B. Taurus* (*Bos Taurus*), *B. bubalis* (*Bubalus bubalis*), *S. meridionalis* (*Silurus meridionalis*), *D. rerio* (*Danio rerio*), *C. idella* (*Ctenopharyngodon idella*), *O. mykiss* (*Oncorhynchus mykiss, S. salar* (*Salmo salar*), T. orientalis (*Thunnus orientalis*), *V. moseri* (*Verasper moseri*), *M. salmoides* (*Micropterus salmoides*), *S. maximus* (*Scophthalmus maximus*), *S. aurata* (*Sparus aurata*), *A. schlegelii* (*Acanthopagrus schlegelii*), *L. japonicus* (*Lateolabrax japonicus*), *D. labrax* (*Dicenthrachus labrax*), *E. coioides* (*Epinephelus coioides*), *L. crocea* (*Larimichthys crocea*), *S. chuatsi* (*Siniperca chuatsi*), *O. mossambicus* (*Oreochromis mossambicus*), *O. niloticus* (*Oreochromis niloticus*), *C. melanopterus* (*Carcharhinus melanopterus*). Length of the branches corresponds to number of substitutions per site and confidence values (based on a bootstrap number of 1,000) are shown above and below the lines, respectively.

**Figure 3:**
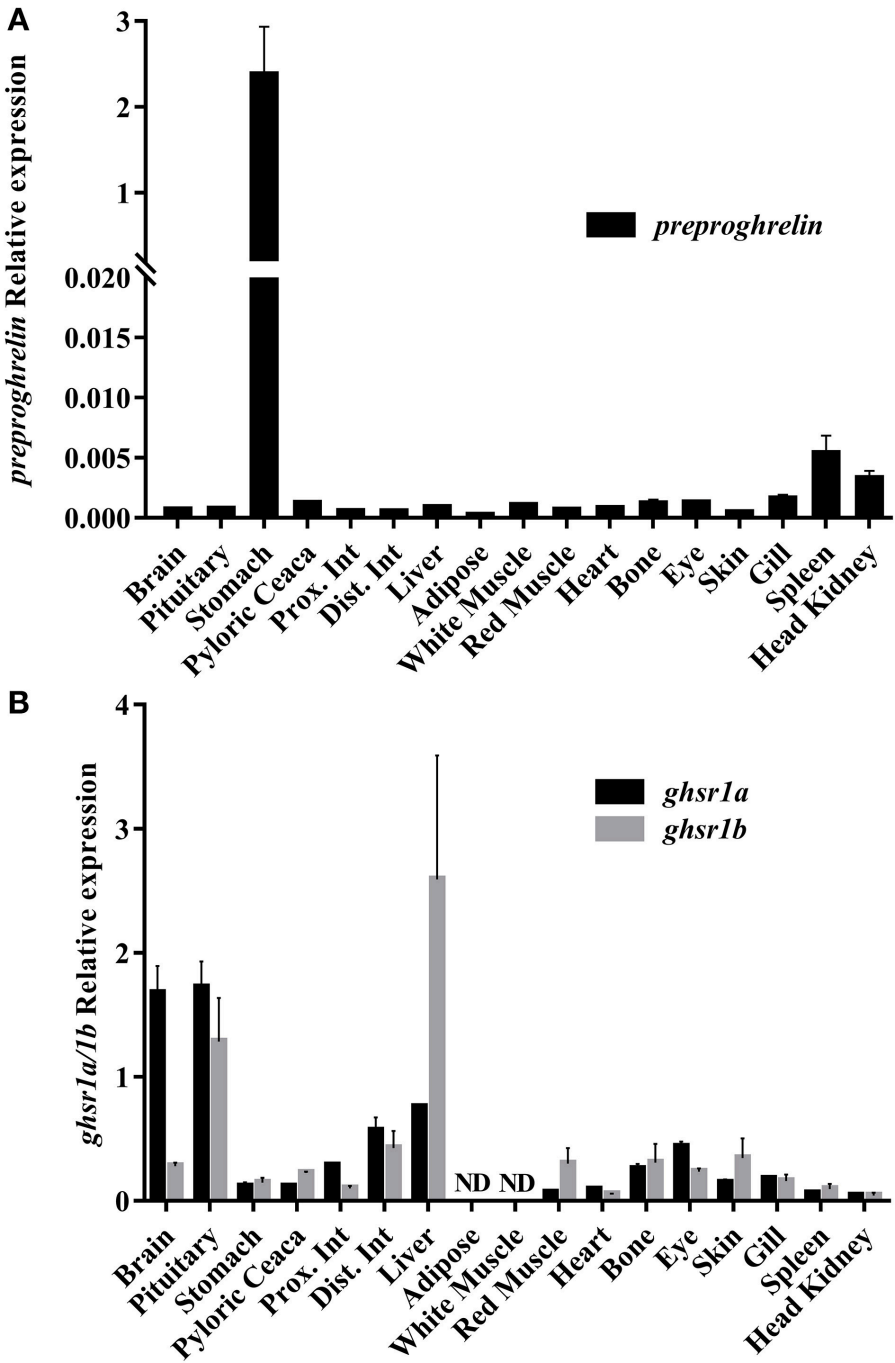
Relative gene expression across a tissue screening of *preproghrelin* **(A)** and Ghrelin receptors *ghsr1a* and *ghsr1b* **(B)**. Data are shown as means ± SEM from three individual fish (*n* = 3). Prox. Int, proximal intestine; Dist. Int, distal intestine.

The phylogenetic analysis of the Preproghrelin amino acid sequence is shown in Figure 2. The unrooted tree highlights the conservation of this protein in vertebrates, although it presents clusters that separate the different vertebrate classes and fish orders. Results of the *preproghrelin* and *ghsrs* gene expression screening are shown in Figures 3A,B, respectively. *preproghrelin* was mainly expressed in stomach, but weak expression was also detected in many other tissues (i.e., spleen and head kidney). Regarding the receptors, brain, pituitary and liver were the tissues with highest expression of both, *ghsr1a* and *ghsr1b*, although low levels of expression were also found in many other. In pituitary and brain, the expression levels of *ghsr1a* were very similar, but the expression of *ghsr1b* was higher in liver. Thus, in pituitary and brain the most abundant isoform was *ghsr1a* while in liver was *ghsr1b*.

### Fasting and refeeding experiment

#### Growth and morphometric parameters

**Figure 4:**
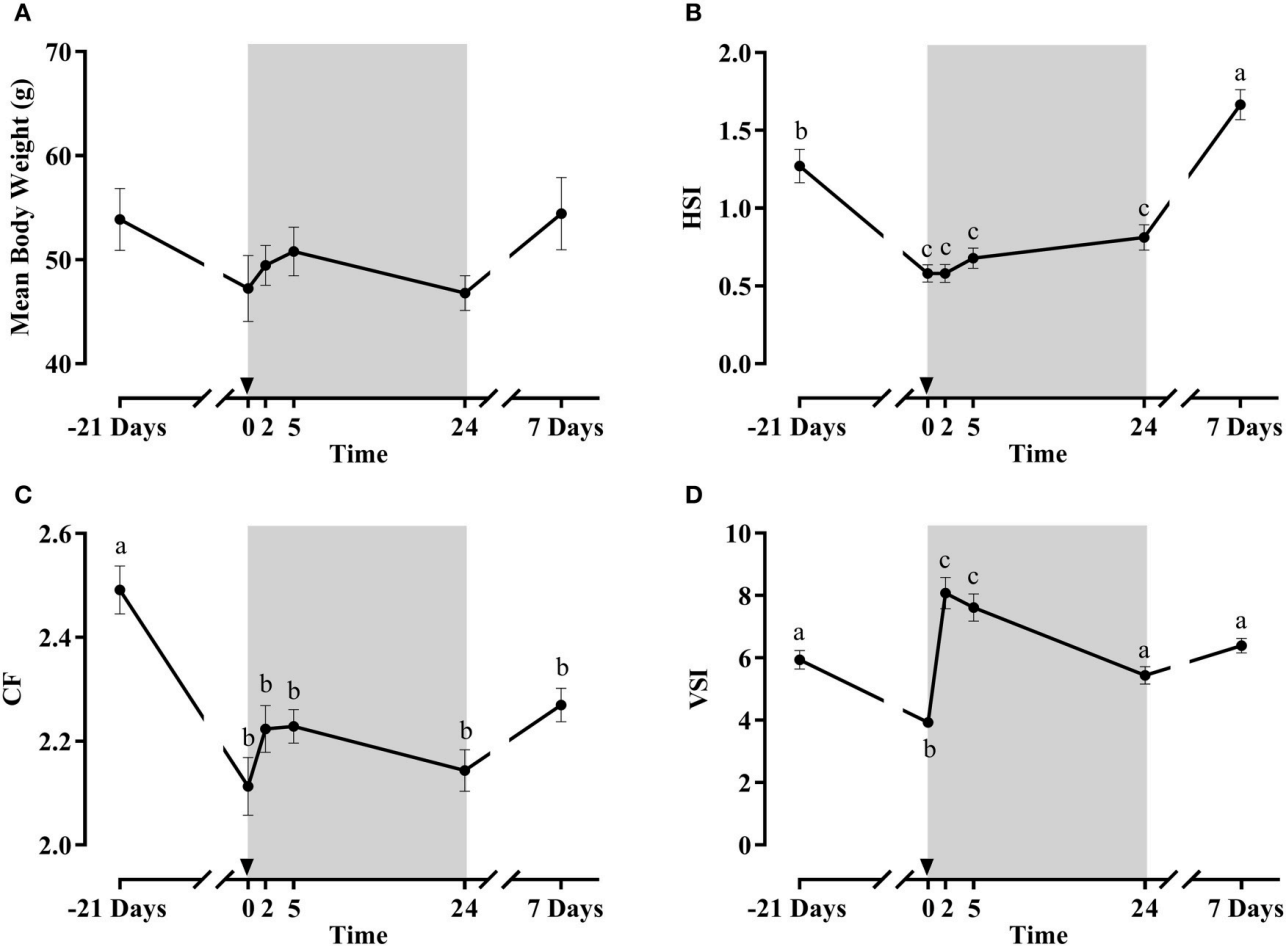
Mean body weight **(A)**, condition factor (CF) **(B)**, hepatosomatic index (HSI) **(C)** and viscerosomatic index (VSI) **(D)** of fish during the fasting and refeeding experiment. The postprandial period is shown in gray boxes and the time in hours. Data are shown as means ± SEM (*n* = 6). Letters indicate significant differences (*p* < 0.05) by one-way ANOVA and Tukey HSD or LSD test.

Morphometric parameters results are shown in Figures 4A–D. Mean body weight (which was not significantly affected) and CF presented a similar pattern along the fasting/refeeding experiment, decreasing after fasting and slightly increasing afterwards, partially recovering at day 7. Regarding HSI, a significant decrease was observed after fasting, but was significantly increased at day 7 post-refeeding. At 2 h post-prandial the stomach was clearly full, but no food was found in the intestine, whereas at 5 h the stomach had emptied almost all its food content. Thus, VSI was significantly lower after the fasting period. With refeeding, it increased at 2 and 5 h, but at 1 and 7 days the VSI values returned to baseline levels.

#### Plasma Ghrelin, Gh and Igf-1

**Figure 5:**
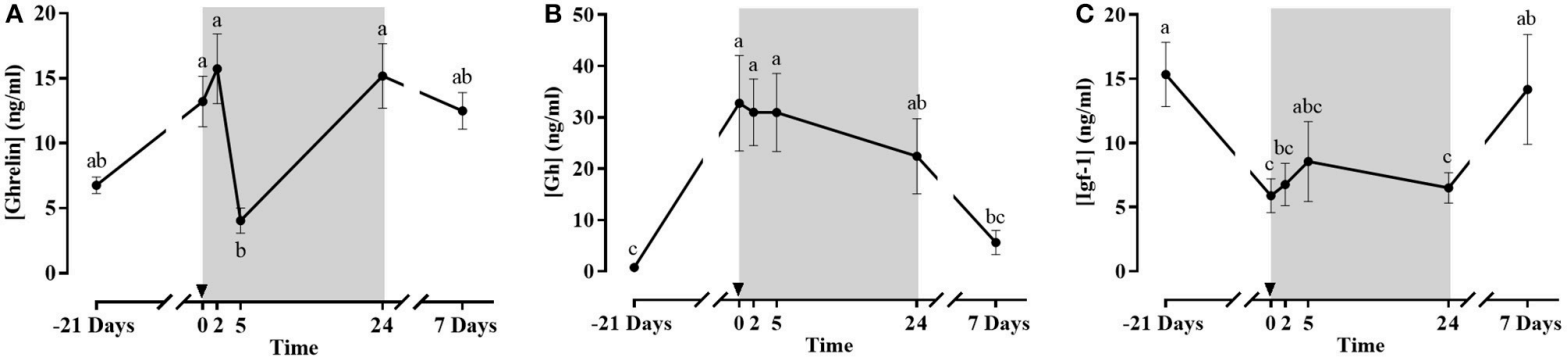
Plasma concentration of Ghrelin **(A)**, Gh **(B)** and Igf-1 **(C)** during the fasting and refeeding experiment. The postprandial period is shown in gray boxes and the time in hours. Data are shown as means ± SEM (*n* = 6). Letters indicate significant differences (*p* < 0.05) by one-way ANOVA and Tukey HSD or LSD test.

Ghrelin, Gh, and Igf-1 plasma concentrations are presented in Figures 5A–C. Plasma Ghrelin showed maximum levels after fasting and at 2 h post-prandial, and a significant dip at 5 h, but then returned to high levels after 1 and 7 days. However, it should be taken into account that those samples, as well as the one before the whole fasting period, were taken after a 24 h fast, which appears to be a potential stimulus for Ghrelin secretion. Circulating Gh increased significantly with fasting. Then, there was no acute post-prandial change but a gradual decrease upon refeeding returning to basal after 7 days. Plasma Igf-1 levels had an inverse pattern to that of Gh; showing significantly lower values after the 21 days fasting period compared to day 0 and then returning to basal levels at 7 days post-refeeding.

#### Gene expression

##### Preproghrelin and ghsrs

**Figure 6:**
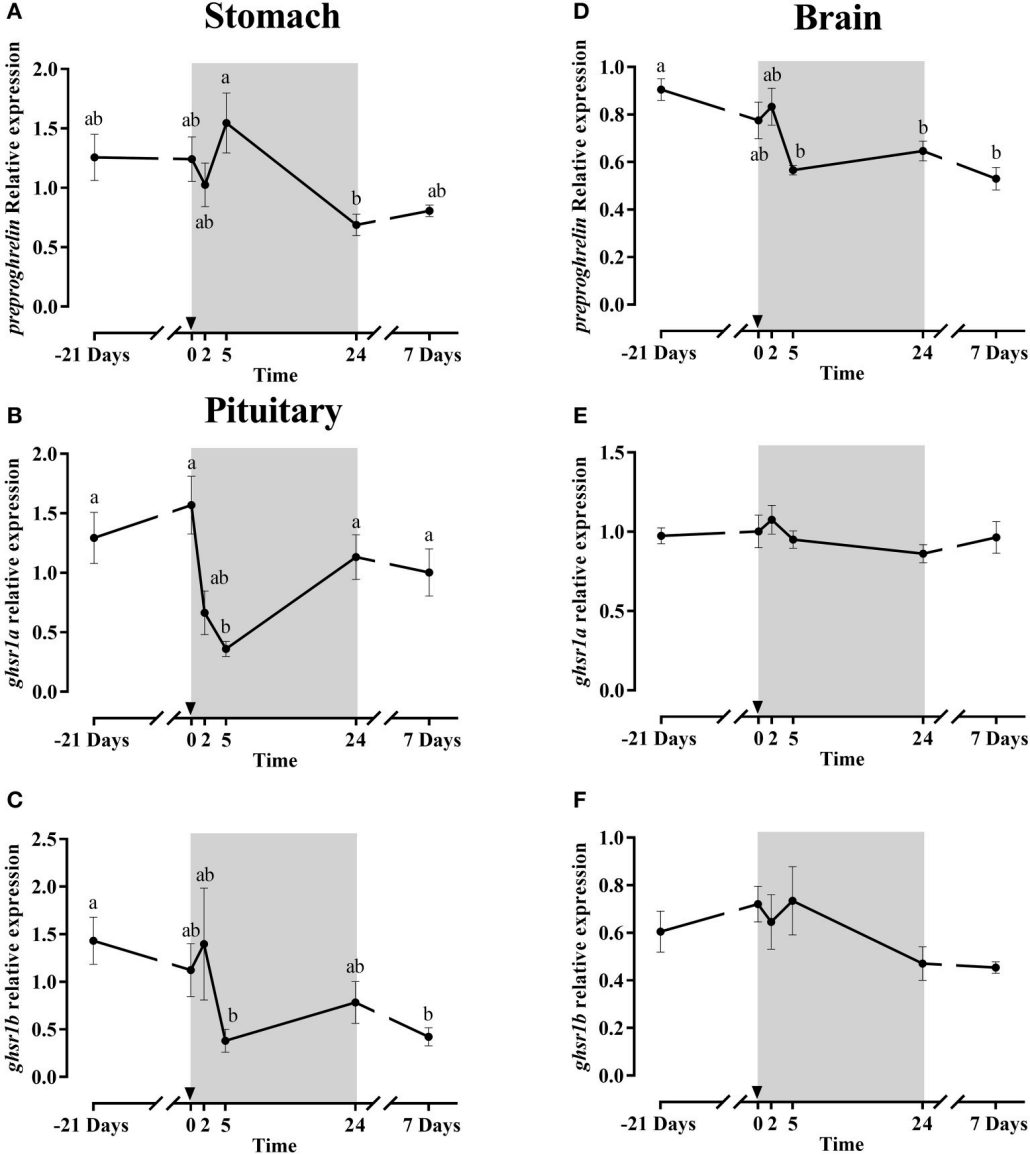
Relative gene expression of stomach *preproghrelin* **(A)**, pituitary *ghsr1a* **(B)** and *ghsr1b* **(C)** and brain *preproghrelin* **(D)**, *ghsr1a* **(E)** and *ghsr1b* **(F)** during the fasting and refeeding experiment. The postprandial period is shown in gray boxes and the time in hours. Data are shown as means ± SEM (*n* = 6). Letters indicate significant differences (*p* < 0.05) by one-way ANOVA and Tukey HSD or LSD test.

Stomach *preproghrelin* gene expression (Figure 6A) did not show any change after fasting, but a significant difference was observed after 1 day of refeeding. In the brain, *preproghrelin* gene expression was much lower than in the stomach (Figure 3A); fasting effects were not found either but at 5 h post-prandial the expression levels in the brain were significantly down-regulated (Figure 6D) compared to the initial sampling (−21 days), and similar low expression values were maintained at 1 and 7 days post-feeding.

The mRNA expression profile of both pituitary *ghsrs* isoforms (Figures 6B,C) was similar along the experiment, stable during fasting and down-regulated significantly at 5 h refeeding. After 1 and 7 days, the expression of *ghsr1a* increased significantly reaching the levels as before fasting, while the *ghsr1b* expression remained low until the end. Moreover, the gene expression patterns of both *ghsrs* in the brain (Figures 6E,F) were almost identical, being practically irresponsive to either 21 days of fasting or the onset of feeding.

##### Gh/Igf-1 axis members

**Figure 7:**
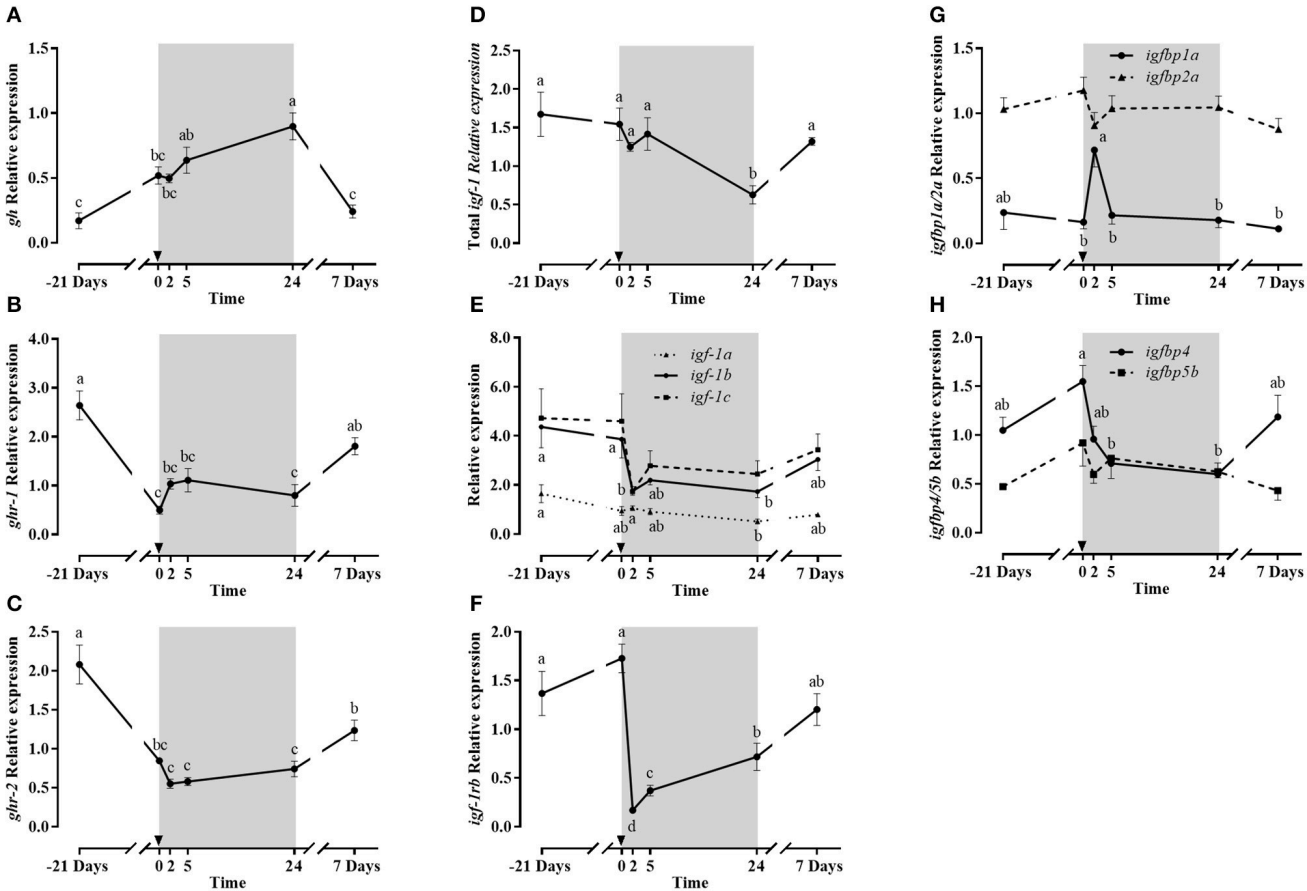
Relative gene expression of pituitary *gh* **(A)**, liver *ghr-1* **(B)** *ghr-2* **(C)** total *igf-1* **(D)**, *igf-1* splice variants **(E)** *igf-1rb* **(F)**, *igfbp1a* and *igbp2a* **(G)** and *igfbp4* and *igfbp5b* **(H)** during the fasting and refeeding experiment. The postprandial period is shown in gray boxes and the time in hours. Data are shown as means ± SEM (*n* = 6). Letters indicate significant differences (*p* < 0.05) by one-way ANOVA and Tukey HSD or LSD test.

Pituitary *gh* gene expression (Figure 7A), similarly to plasma Gh, progressively increased to reach maximum levels at 1 day post-refeeding, decreasing back to basal levels at day 7.

The liver gene expression of total *igf-1* (Figure 7D) remained stable after fasting and in the early post-prandial period, but after 1 day, the lowest levels were observed, and at day 7 returned to baseline. The *igf-1* splice variants (Figure 7E) showed a similar gene expression profile than that of total *igf-1*, especially *igf-1a* with little effects of fasting and lowest expression levels at 1 day post-refeeding, recovering basal values after 7 days. Moreover, *igf-1b* and *igf-1c* showed a significant post-prandial dip at 2 h, maintaining still lower values at day 1, to return to basal levels at day 7.

Concerning liver Gh receptors, both were significantly down-regulated due to fasting (Figures 7B,C). However, different post-prandial responses were observed: *ghr-1* stopped decreasing at 2 and 5 h, while *ghr-2* expression continued to decline until 2 h, remaining low up to 1 day post-refeeding. The expression of both receptors was then up-regulated at day 7 in comparison to early post-prandial measurements. In the case of Igf-1 receptors, the only isoform detected in liver was *igf-1rb* (Figure 7F). Its expression was not affected by fasting but was significantly down-regulated at 2 h of refeeding, to then recover at 7 days initial expression levels.

The gene expression of four *igfbps* is shown in Figures 7G,H. *igfbp1a* and *igfbp2a* expression had similar stable patterns, except that *igfbp1a* showed a significant abrupt peak in expression 2 h post-refeeding, returning to basal levels at 5 h. The expression of *igfbp4* and *igfbp5b* was detected for the first time in gilthead sea bream liver. Both presented a similar profile, but *igfbp5b* did not show significant changes while the response for *igfbp-4* was more pronounced, with a significant decrease at 5 h and 24 h post-prandial compared to the onset of refeeding. Then, such low expression level was maintained after 1 day post-refeeding and basal levels were recovered after 7 days.

## Discussion

### Preproghrelin and ghsrs characterization

Since its discovery, the *preproghrelin* nucleotide and amino acid sequences have been described in many vertebrate species (36). In the present study, phylogenetic analysis of the gilthead sea bream translated sequence highlighted the conservation of the most characteristic features. In fact, Preproghrelin is considered a well-conserved protein, but with a perceptible evolution among classes and orders. The gilthead sea bream Preproghrelin resulted more closely related to other Sparidae species, flatfishes and European sea bass (*Dicentrachus labrax*), but more distant to salmonids, cypriniformes, siluriformes and chondrictyes.

The expression of *preproghrelin* was detected mainly in stomach and pyloric caeca, which agrees with previous studies in mammals and other fish species, establishing that the main source of Ghrelin is the stomach (3, 4, 6, 12, 13). Moreover, weak *preproghrelin* expression was detected in other tissues and organs as in different fish species (5, 6). One of the main targets of Ghrelin is the brain, where it is reported to act in appetite-regulating areas to induce (or decrease in some species) feed intake (19, 22). Thus, the detection of *preproghrelin* mRNA expression locally in the brain may also contribute to confirm the existing hypothesis that Ghrelin is synthetized both peripherally and centrally (22). In our screening, the low *preproghrelin* mRNA levels detected in the brain may be due to the fact that the whole brain was taken, instead of only the hypothalamus, which is supposed to be the main production site and target in the brain.

The gene expression screening of the two *ghsrs* showed that both are widely distributed among multiple tissues and organs, in line with previous research (16). The tissues with higher expression were pituitary, brain and liver, which support that these are the main targets of Ghrelin action in gilthead sea bream, as in many other vertebrate species (15, 37). Furthermore, as far as we know, this is the first time that it is observed that isoform *a* is more abundant in brain and pituitary, while isoform *b* is more abundant in liver. Such differential expression in these tissues could suggest that Gh secretion requires the presence of the truncated isoform to achieve better regulation, as suggested (15, 16).

### Fasting and refeeding effects on growth performance and ghrelin

Although gilthead sea bream tolerates long periods of food deprivation well (25, 38–40), the morphometric parameters reduction after 21 days of fasting confirmed that the fish had entered in a catabolic state, which was progressively reverted upon refeeding, as demonstrated by the recovery of the body indexes at the end of the experiment.

The existing literature reveals that the response of Ghrelin to fasting may be, especially in fish, species-specific. Thus, fasting has been reported to up-regulate, down-regulate or unchange the gastrointestinal tract and brain *ghrelin* mRNA levels in diverse fish species (7, 41–45). Such a variety of responses could indicate that other factors, such as sex and age of individuals (44), temperature (46), fasting duration (42) or diet (13) may also affect Ghrelin production. Interestingly, during the development of the present work, Babaei et al. (13) also reported a tissue-specific *preproghrelin* expression response to fasting in gilthead sea bream.

The different response observed to 21 days of fasting with Ghrelin plasma levels and *preproghrelin* mRNA levels in stomach, is consistent with previous fish studies (45, 47) and suggests that post-transcriptional mechanisms are in place. However, Ghrelin plasma levels were also high at 1 and 7 days post-refeeding probably due to the 24 h fast. In sea bass, a rise in *preproghrelin* expression was observed during the first days of fasting, to then decrease progressively to fed control values after 21 days of fasting (48). In grass carp, a peak of intestinal *ghrelin* expression was described after 7 days of fasting (49). In goldfish, Unniappan et al. (42) found that fasting for 3 and 5 days significantly increased Ghrelin plasma levels, while in gut or hypothalamus *preproghrelin* expression did not increase until after 7 days of fasting. Moreover, in Atlantic salmon, Ghrelin levels were significantly increased after 2, but not 14 days of fasting (50). Together, these observations support the idea that in diverse fish species, the response increasing Ghrelin plasma levels occurs mainly during the early stage of fasting and is not always related to changes in gut gene expression.

Besides, with refeeding Ghrelin plasma levels that were still high at 2 h, were followed by a significant decrease at 5 h, suggesting an inhibitory effect on Ghrelin secretion as food enters the stomach. These decrease in Ghrelin coincided with the beginning of circulating Gh decline, suggesting the relationship between these two hormones. A similar decrease was also observed at 1 h post-prandial in tilapia (51), and in refed striped bass (52). Moreover, such reduced plasma levels coincided with the peak in stomach *preproghrelin* mRNA levels, whereas the minimum expression 1 day after refeeding corresponded with the recovery of Ghrelin plasma levels, indicating an inverse relationship between the regulation of the gene expression and the circulating hormone. Thus, it appears that during this specific postprandial stage (2, 5, and 24 h) *preproghrelin* gene expression could be regulated by Ghrelin plasma levels.

Unniappan et al. (42) also observed that in goldfish, *preproghrelin* mRNA levels (in gut and hypothalamus) and Ghrelin plasma levels were sensitive to feeding when analyzed periprandially. At 3 h pre-meal, Ghrelin plasma and mRNA levels were high, and 1 and 3 h after feed intake were down-regulated in both tissues. Similar results were observed by Hatef et al. (53) in zebrafish, in which *preproghrelin* mRNA levels in brain and gut were down-regulated 3 h post-meal and increased in fasted fish. These studies are in accordance with the observed decrease in plasma Ghrelin and brain *preproghrelin* mRNA at 5 h post-feeding in the present experiment, indicating that Ghrelin may be mainly regulated by feed intake also in gilthead sea bream.

### Fasting and refeeding effects on ghsrs

The *ghsrs* responded differentially to refeeding in brain and pituitary. The expression in brain remained constant, while in the pituitary decreased progressively up to 5 h to recover at 1 or 7 days of refeeding the expression of *ghsr1a*, and to a lesser extent of *ghsr1b*. In rats, brain and pituitary *ghsrs* were up-regulated in fasting and decreased after refeeding (15, 54, 55). However, the function of Ghsrs in fish and other non-mammalian vertebrates is still not fully understood. Thus, although Ghsrs have crucial roles in the ghrelinergic system and their expression is finely regulated by nutritional condition, hormonal status and environmental factors, their response is highly variable depending on the species especially in fish, in which a higher number of Ghsrs isoforms has been described (19).

Peddu et al. (51) did not find in Mozambique tilapia brain a clear response to fasting in *ghsrs* expression, but at feeding time (just before food administration) both receptors were up-regulated to decrease at 1 and 3 h post-feeding. In the same species, a significant change was not observed in brain *ghsr1a* expression between 1 and 7 days of fasting, while *ghsr1b* increased after 3 but not 5 fasting days (56). In Atlantic salmon, a fasting period of 2 or 14 days did not change *ghsr1a* brain expression (50), neither it did 15 days of fasting in zebrafish *ghsrs* (57). Contrarily, Kaiya et al. (58) found that 7 days of fasting induced a decrease in the expression of *ghsr1a* in the vagal lobe of goldfish. Thus, although species differences exist it seems that there is regulation of *ghsrs* depending on the alimentary condition.

Ghrelin receptors in fish pituitary have been poorly investigated, but low basal expression levels have been found in tilapia (56), goldfish (59, 60) or yellow catfish (61). In the case of grass carp, 14, 21, and 28 days of fasting resulted in increased pituitary gene expression of *ghsr1a* that correlated with increased plasma Gh and *preproghrelin* pituitary gene expression (62). Moreover, these authors found that Ghrelin administration provoked an increase in pituitary *ghsr1a* expression. In the present study, the decrease in *ghsrs* expression during the post-prandial stage was noticeable and related with circulating Ghrelin, pointing to a slowdown of the system during food intake. To summarize, Ghrelin receptors expression in the brain do not show a uniform regulation among fish species and seem to be less influenced by the nutritional condition in comparison to mammals. Furthermore, less is known about pituitary Ghsrs dynamics during fasting in fish, but in gilthead sea bream, both isoforms present a similar response that parallels Ghrelin plasma levels.

### Fasting and refeeding effects on the Gh/Igf axis

The rise of circulating Gh during fasting was parallel to *gh* mRNA levels in the pituitary, being significantly high at 5 h post-feeding. The expression of *gh* remained high until 1 day of refeeding, and similarly to plasma Gh, returned to basal values after 7 days, thus indicating the important and extended effect of fasting in this hormone. This response of Gh to fasting and refeeding has been observed in previous studies in various fish species, such as Chinese perch (*Siniperca chuatsi*), tilapia and black sea bream (*Spondyliosoma cantharus*) (63–65). Plasma Igf-1 also responded to nutritional state, presenting an inverse pattern to that of Gh, decreasing with fasting and slowly increasing with refeeding. Liver total *igf-1* gene expression as well as its splice variants partially recovered after 7 days of refeeding. These results are in line with previous works (63, 66). The inverse correlation between Gh and Igf-1 plasma levels during fasting was pointed out in gilthead sea bream previously (38, 67, 68), and has been described in several other fish species (e.g., coho salmon, chinook salmon, channel catfish, Nile tilapia or gilthead sea bream) in diverse conditions (26, 47, 66, 69–71). Moreover, the results support that the circulating Gh/Igf-1 ratio is a good indicator of metabolic state in gilthead sea bream and that it is clearly affected by feeding condition (67, 72). Picha et al. (52) suggested that during fasting in striped bass, high Ghrelin levels contribute to counteract the negative feedback normally exerted by Igf-1 on Gh release, in order to maintain Gh secretion.

The gene expression of *ghrs* in the liver also reflected the nutritional status. The dramatic down-regulation of both *ghr-1* and *ghr-2* expression, along with increased Gh plasma levels, suggests a Gh liver desensitization during the fasting period (23). After refeeding, a rapid increase in the mRNA levels of *ghr-1*, the isoform mostly related with anabolic processes in this species was observed, and later in the expression of *ghr-2*, indicating that ingested nutrients may have initiated growth promotion (23). Furthermore, liver *igf-1rb* showed a similar tendency to that of *ghrs* after refeeding and its abrupt post-prandial expression drop at 2 h was not recovered until the end of the trial. It is interesting that this response is parallel to *igf-1b* and *c* hepatic gene expression. Down-regulation of liver *igf-1rb* expression was also observed in gilthead sea bream during exercise (26), but as far as we know, this is the first time that this effect is found in refed fish.

The expression of *igfbps* was stable during fasting while 7 days of refeeding recovered their basal values. Nevertheless, *igfbp-4* presented the highest expression after 21 fasting days in agreement with its Igf-1 conservative function, while the increase of *igfbp-1a* at 2 h post-feeding fitted well with its recognized role in mobilization conditions in this species. Similarly, in a fasting and refeeding experiment in rainbow trout, Gabillard et al. (73) observed different responses for *igfbps*. Hevrøy et al. (50) described the effects of fasting on Ghrelin and Gh/Igf-1 system in Atlantic salmon, in which Igfbp-1 seemed to be a marker of catabolic state. Breves et al. (63) demonstrated different roles of *Igfbps* during fasting, and indicated that Igfbp-1b may operate to reduce Igf-1 signaling during fasting in tilapia. The functional relationship between Gh, Igf-1 and Ghrelin during fasting in fish needs to be further investigated.

To summarize, the full *preproghrelin, ghsr1a and ghsr1b* nucleotide sequences and their response during fasting/refeeding have been described for the first time in gilthead sea bream. Both, long term (21 days) and short term (24 h) fasting increased circulating Ghrelin, which showed the lowest values few hours post-prandial. The plasma Ghrelin dip was also reflected by pituitary *ghsrs*, suggesting that Ghrelin's stimulatory action on Gh secretion is modulated by feeding. Plasma Gh levels were elevated in parallel with its pituitary gene expression returning to basal levels after 7 days of refeeding, although at this time circulating Ghrelin was again increased. Taken together, the data suggest that Ghrelin can be a regulator of Gh secretion in gilthead sea bream, but the metabolic state itself and other regulatory molecules may exert important effects. Finally, this study indicates that in gilthead sea bream, Ghrelin secretion is mainly related to the progress of the digestive process, showing a down-regulation in the post-prandial period to rise again just before feeding.

## Author contributions

MP-A, EV, and JG conceived and designed the experiments; MP-A, EV, JV-A, AS-M, NR-H, JF-B, JB, IN, EC, and JG performed the experiments; MP-A, EV, JV-A, AS-M, NR-H, IH, JF-B, JB, JC-G, IN, EC, EJ, JP-S, and JG analyzed the data and interpreted the results; JF-B, JB, IN, EC, EJ, JP-S, and JG contributed reagents, materials, analysis tools; MP-A, EV, JV-A, AS-M, NR-H, IH, JF-B, JB, JC-G, IN, EC, EJ, JP-S, and JG wrote and revised the paper.

## Data availability statement

The three sequences obtained in the present study are deposited in GenBank (NCBI) under accession numbers: MG570187 for *preproghrelin*; MG570188 for *ghsr1a*, and MG570189 for *ghsr1b*. All relevant data is contained within the manuscript.

### Conflict of interest statement

The authors declare that the research was conducted in the absence of any commercial or financial relationships that could be construed as a potential conflict of interest.
