# Supplementary material for: Ghrelin and Its Receptors in Gilthead Sea Bream: Nutritional Regulation
Source: Front Endocrinol (Lausanne). 2018 Jul 30;9:399. doi: 10.3389/fendo.2018.00399 (PMC6077198; doi:10.3389/fendo.2018.00399)
Supplement: Supplementary file 1 [file Image_1.pdf]

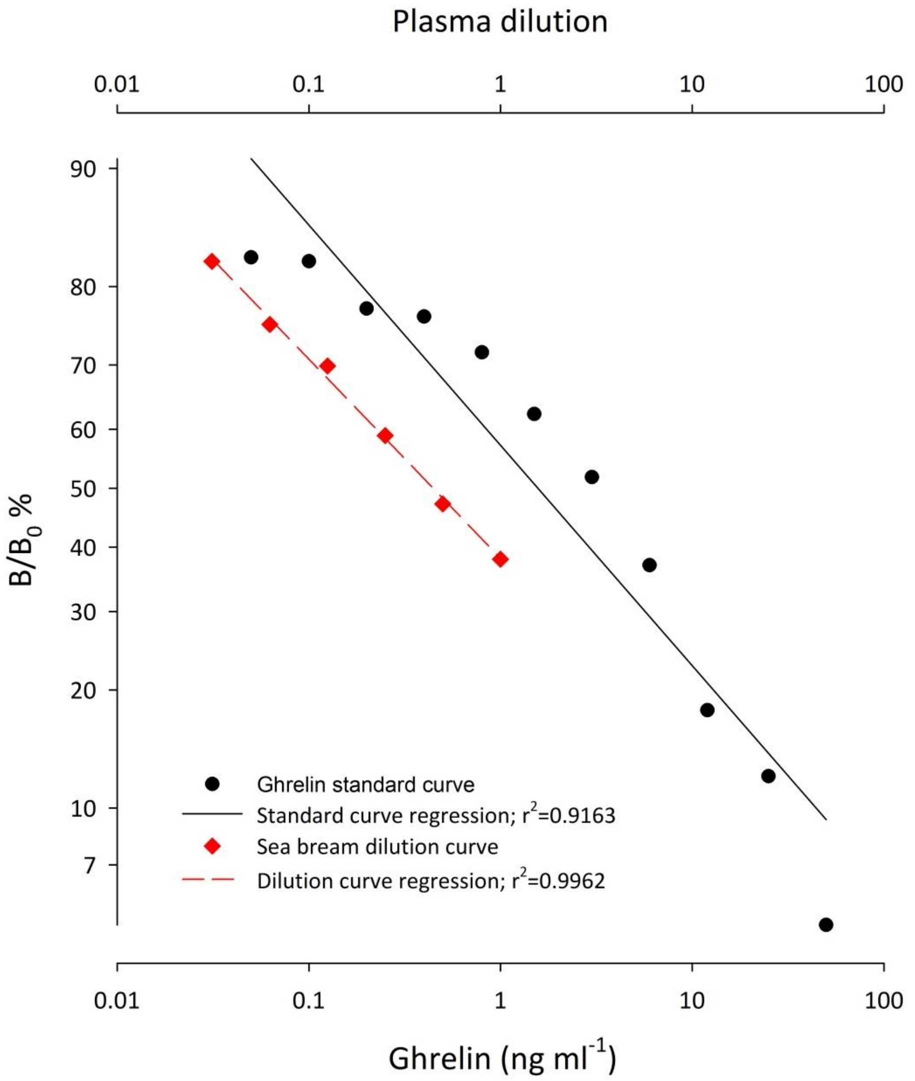

Supplementary figure 1. Binding inhibition curves on a log-logit scale showing parallel specific binding for synthetic rainbow trout Ghrelin standard (filled circles) and serial dilutions of sea bream plasma (squares) to the anti-rat Ghrelin [1-11] antisera.
